# Validation of the cross-cultural dementia screening test in Alzheimer’s disease and Parkinson’s disease

**DOI:** 10.3389/fpsyg.2022.1043721

**Published:** 2023-01-04

**Authors:** Alfonso Delgado-Álvarez, Cristina Delgado-Alonso, Miriam Goudsmit, Rocío García-Ramos, María José Gil-Moreno, María Valles-Salgado, María Díez-Cirarda, María Dolores Zamarrón-Cassinello, Jorge Matías-Guiu, Jordi A. Matias-Guiu

**Affiliations:** ^1^Department of Neurology, Hospital Clinico San Carlos, San Carlos Institute for Health Research (IdiSSC), Universidad Complutense de Madrid, Madrid, Spain; ^2^Faculty of Psychology, Universidad Autónoma de Madrid, Madrid, Spain; ^3^Department of Psychiatry and Medical Psychology, OLVG Hospital, Amsterdam, Netherlands

**Keywords:** Alzheimer’s disease, cognitive screening, cross-cultural neuropsychology, mild cognitive impairment, Parkinson’s disease

## Abstract

**Objective:**

The Cross-Cultural Dementia (CCD) is a new screening tool to evaluate cognitive impairment based on a cross-cultural perspective to reduce the bias of education, and language and cultural differences. We aimed to evaluate the diagnostic properties of the CCD in Spaniards for the assessment of patients with Alzheimer’s disease in mild cognitive impairment (AD-MCI) and mild dementia stages (AD-D) and patients with mild cognitive impairment associated with Parkinson’s disease (PD-MCI).

**Methods:**

Sixty participants with AD (50% MCI) and thirty with PD-MCI were enrolled. Each clinical group was compared against a healthy control group (HC) with the same number of participants and no significant differences in age, education, and sex. A comprehensive neuropsychological test battery and CCD were completed. Intergroup comparisons, ROC curves, and cut-off scores were calculated for the study of diagnostic properties.

**Results:**

Intergroup differences were found in accordance with the cognitive profile of each clinical condition. Memory measures (Objects test) were especially relevant for the classification between AD and HC. Memory and executive function scores (Sun-Moon and Dots tests) were useful in the case of PD-MCI and HC. Furthermore, CCD described differences in executive functions and speed scores comparing AD-MCI and PD-MCI. Correlations between standardized neuropsychological tests and CCD measures supported the convergent validity of the test.

**Conclusion:**

CCD showed good discrimination properties and cut-off scores for dementia and extended its application to a sample of prodromal stages of AD and PD with mild cognitive impairment.

## 1. Introduction

Globalization has resulted in multicultural and diverse spaces where different languages, cultures, and education systems coexist. In Europe, different migratory movements inside and outside the continent have taken place, showing great diversity nowadays (Franzen et al., 2021). In this regard, different research groups highlighted the need for more cross-cultural measures and validation studies, especially screening tools, to assess different clinical groups in Europe (Franzen et al., 2022a).

The study of culture and its meaning in neuropsychological assessment has focused on the description of different variables correlated with culture, such as patterns of ability, familiarity, cultural value, acculturation, and language (Ardila, 2007; Rosselli et al., 2022). In this regard, education, which is strongly associated with patterns of ability, and language are especially important during the cognitive assessment. Thus, different cross-cultural instruments have been developed trying to minimize the effects of culture in neuropsychological assessments.

In Alzheimer’s disease (AD), as the most common cause of dementia (Mayeux and Stern, 2012), some cross-cultural tools have been recently described (Goudsmit et al., 2017; Nielsen et al., 2019; Franzen et al., 2022a,b) as an alternative to classic cognitive screening tests that show differential item functioning (Jones and Gallo, 2002). The most common symptoms of AD at early stages are cognitive impairment, where episodic memory deficits play the most significant role, and changes in functioning and behavior (Dubois et al., 2016). From a cross-cultural perspective, RUDAS has shown important advantages (Nielsen and Jørgensen, 2020), compared with the traditional screening test Mini-Mental State Examination test (Nielsen et al., 2012; Goudsmit et al., 2018). However, due to the verbal load of RUDAS, an interpreter could be necessary to correctly apply the test, which could be a limitation in some settings.

While some advances have been reported in the field of AD, fewer studies have investigated Parkinson’s disease (PD), the second most frequent neurodegenerative disorder and a common cause of cognitive impairment (Poewe et al., 2017). The main clinical characteristic of PD is motor disorders. However, cognitive deficits are also frequent, including executive functioning, attention, visuospatial abilities, and memory deficits (Muslimovic et al., 2005). The Montreal Cognitive Assessment (MoCA) and Scales for Outcomes in Parkinson’s Disease-Cognition (SCOPA-COG) have been recommended as screening tests for PD with mild cognitive impairment. However, a lack of cross-cultural screening tests has been underscored in this pathology (Skorvanek et al., 2018; Statucka et al., 2021).

Recently, the Cross-Cultural Dementia screening (CCD), a novel neuropsychological dementia screening test, has been developed. It has shown good psychometric properties in previous studies of dementia (Goudsmit et al., 2017). CCD consists of three subtests: Objects test, Sun-Moon test, and Dots test, to measure memory, mental speed, and executive function in people with little or no education and in multicultural settings. Some of the most important advantages of CCD, compared to other screening tests, are the short administration time, the assessment of different cognitive domains, the low verbal load, and the cross-cultural approach, including recorded instructions in different languages to perform the test with a minimal impact of mother tongue on the scores. For these reasons, CCD may be a valuable cognitive screening test (Matias-Guiu and Delgado-Álvarez, 2022). However, a validation process is always required before the clinical practice implementation.

The CCD was validated in a sample of 54 participants (43% Alzheimer’s disease, 19% Alzheimer’s disease and vascular dementia, 17% vascular dementia, 16% dementia not otherwise specified, 3% fronto-temporal dementia, 2% Lewy body dementia) in the Netherlands (Goudsmit et al., 2017). This sample included patients from the Netherlands, Turkey, Morocco, and Suriname. The test was also well-tolerated as part of the TULIPA battery (Franzen et al., 2022a,b). Furthermore, CCD has been regarded as a promising cognitive test for the study of the prevalence of mild cognitive impairment and dementia in Non-Western immigrants, according to a study in a multi-cultural sample of 2,254 participants conducted in the Netherlands (Parlevliet et al., 2016).

To our knowledge, there are no studies using the CCD beyond the Netherlands. At the same time, Hispanic culture is a broad culture that coexists in different continents with different cultures and has its own linguistic and cultural issues (Ardila, 2003). Thus, we aimed to evaluate the diagnostic properties of the CCD in Spaniards. Previous studies have validated CCD as a tool for dementia and have not explored its utility to draw cognitive profiles associated with different neurological diseases. As a novelty, we focused on its diagnostic properties for the assessment of patients with a diagnosis supported by biomarkers of AD in mild cognitive impairment and mild dementia stages, and PD patients with mild cognitive impairment (PD-MCI). In addition, we compared the performance on CCD between AD mild cognitive impairment and PD-MCI.

## 2. Materials and methods

### 2.1. Participants

One hundred and fifty participants were recruited at the Department of Neurology of Hospital Clínico San Carlos in Madrid. Thirty participants with AD – Clinical Dementia Rating CDR = 0.5 (AD-Mild Cognitive Impairment, AD-MCI), 30 with AD – CDR = 1.0 (AD-Dementia, AD-D), 30 with PD mild cognitive impairment (PD-MCI), and 60 healthy controls (50% for comparisons with AD, 50% for comparisons with PD-MCI). Due to the demographic differences between participants with AD and PD-MCI, two HC groups were considered for comparison. There were no statistically significant differences in sex, age, or years of education between each clinical group and its HC group (Supplementary material 1). All participants were Caucasians, Spaniards, and monolinguals (Spanish as their mother tongue). The main demographic and clinical characteristics are shown in Table 1.

**Table 1:** Main demographic and clinical characteristics of all groups.

	AD and HC			PD and HC	
	AD-MCI	AD-D	HC	MCI	HC
N	30	30	30	30	30
Sex, female %	73.3%	73.3%	60%	23.3%	46.7%
Age, years	76.20 (5.85)	76.63 (5.56)	77.37 (5.22)	70.33 (8.68)	67.67 (10.57)
Education, years	7.10 (2.75)	7.03 (3.38)	6.83 (3.86)	11.20 (4.76)	8.90 (4.21)
GDS	0.33 (0.76)	0.73 (0.94)	0.20 (0.66)	0.72 (0.92)	0.13 (0.57)
FAQ	4.33 (3.54)	10.96 (6.34)	0	–	0
IDDD	37.10 (4.24)	42.96 (4.71)	33.87 (1.33)	–	–
SCOPA-COG	–	–	–	20.43 (6.21)	–
Hoehn & Yahr	–	–	–	2.05 (0.34)	–
Impaired memory %	100%	100%	–	63.4%	–
Attention and WM	13.3%	50.0%	–	36.7%	–
Impaired E.F. %	26.7%	46.7%	–	36.7%	–
Impaired language %	10.0%	30.0%	–	3.3%	–
Impaired visuo. %	3.3%	30.0%	–	13.3%	–

GDS, Geriatric Depression Scale; FAQ, Functional Activities Questionnaire; IDDD, Interview for Deterioration in Daily living activities in Dementia; Hoehn & Yahr, Hoehn and Yahr scale; WM, working memory; E.F., executive functions; visuo., visuospatial skills. Impaired domain was based on NN scores, considering a scaled score ≤ 5 as deficit score and impaired domain as ≥ two deficit scores related to the domain (Supplementary material 2).

For AD, inclusion criteria were as follows: (1) complaints of memory loss, (2) biomarkers supporting the diagnosis of AD (temporoparietal hypometabolism in FDG-PET and/or altered A-beta 1–42, tau and phosphotau levels in cerebrospinal fluid), (3) CDR = 0.5 (i.e., memory box = 0.5 and no interference with activities of daily living based on community affairs, home and hobbies, and personal care boxes = 0 for the inclusion of AD-MCI participants), (4) CDR = 1.0 (i.e., memory box ≥0.5 and presence of interference with activities of daily living for AD-D participants), (5) confirmation of clinical progression during the follow-up (Albert et al., 2011). For PD-MCI, inclusion criteria were: (1) diagnosis of PD-MCI according to Movement Disorder Society (MDS), and (2) presence of mild cognitive impairment following MDS criteria Level II (comprehensive assessment) assessing: attention and working memory, executive, language, memory, and visuospatial functions (Litvan et al., 2012; Supplementary material 2). Exclusion criteria for both clinical groups were as follows: (1) prior history of medical, neurological, or psychiatric comorbidity that could bias cognitive assessment, (2) physical difficulties (e.g., hearing or visual problems) with a negative impact on test performance, and (3) scores on MDS-Unified Parkinson’s Disease Rating Scale motor section >30, corresponding to moderate/severe stages (Martinez-Martin et al., 2018).

All HC participants had CDR = 0 and absence of functional impairment assessed by Functional Activities Questionnaire (FAQ) scores = 0 (Olazarán et al., 2005). Furthermore, exclusion criteria were: (1) prior or current history of neurological or psychiatric disease, (2) physical limitations (e.g., hearing or visual problems) with a potential impact on test performance with a potential impact in test performance, and (3) any medical disorder potentially associated with cognitive impairment.

### 2.2. Neuropsychological assessment

Participants with AD or PD-MCI completed the comprehensive neuropsychological battery Neuronorma (NN) (Supplementary material 3), which has normative data in our setting (Peña-Casanova et al., 2009) and the Geriatric Depression Scale (GDS) (Yesavage et al., 1982). In addition, CDR was used to differentiate between AD-MCI (CDR 0.5) and AD-D (CDR 1.0). FAQ and Interview for Deterioration in Daily living activities in Dementia (IDDD) (Böhm et al., 1998) were also administered in the AD group. SCOPA-COG and Hoehn and Yahr scale (Hoehn and Yahr, 1967) were used for cognitive and functional disability staging in the PD-MCI group. HC groups were evaluated through FAQ, CDR, and GDS.

All participants completed the Cross-Cultural Dementia Screening test (Goudsmit et al., 2017), which was not used for diagnosis. CCD is composed of three subtests: Objects test, Sun-Moon test, and Dots test. During Objects test Part A, different colored pictures of everyday objects are presented for immediate (Part A) and delayed (Part B) recognition. Scores on Objects tests are the result of true positives (30) + true negatives (92) with a maximum score of 122.

Sun-Moon test is based on the Stroop interference paradigm. Part A is a naming task, and participants are asked to name two kinds of pictures: sun or moon, as fast as possible. Part B is an interference task, and participants are asked to say the opposite name of the picture (e.g., if they see a sun, they should say “moon”). Time in seconds and errors are recorded.

**Figure 1:**
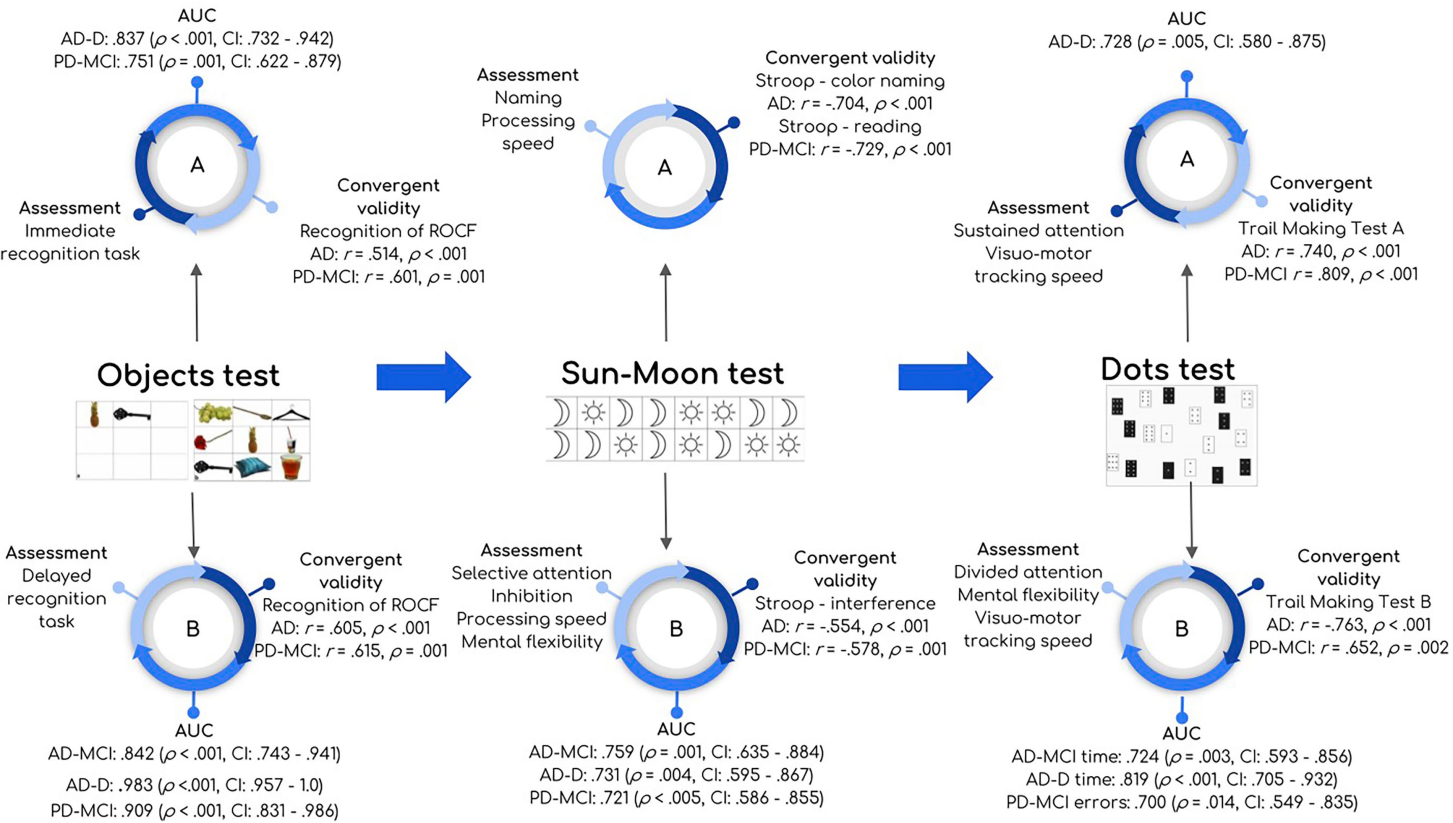
Summary of main results and characteristics of CCD. Figure shows part A and B of Objects test, Sun-Moon test, and Dots test. Each part (A and B) shows: cognitive process assessed (“Assessment”), ROC analysis for each clinical group (“AUC”) describing AUC (value of *p*, confidence interval), and correlations with standard tests (“Convergent validity”) describing *r*, value of *p*. ROC analysis was reported only in those cases with AUC > 0.70 and significant.

Dots test is based on a similar paradigm to the Trail Making Test. During Part A. Participants are asked to connect dominos-like pieces by ascending order, while Part B is composed of black and white pieces to connect and to alternate in ascending order (e.g., 1 white, 1 black, 2 white, 2 black…). Time in seconds and errors are measured. Examples of CCD stimuli are shown in Figure 1.

For the study of convergent validity, the Free and Cued Selective Reminding Test (FCSRT), Rey Osterrieth Complex Figure (ROCF) (recall after 3 min, 30 min, and recognition tasks), Trail Making Test (TMT), and Stroop test were used as convergent measures, in accordance with the cognitive processes underlying CCD subtests.

### 2.3. Procedure

The study was conducted with the approval of our hospital’s Ethics Committee (code 19/126-E), and all participants gave written informed consent.

Trained neuropsychologists carried out the neuropsychological assessment in two sessions with a total duration of 3 h. Neuropsychologists administered the task verbally. In PD-MCI, NN scores confirmed the condition of mild cognitive impairment (at least two age-and education-adjusted scaled scores ≤5 in one or more cognitive domains), and participants were tested in their optimal motor stage, according to MDS recommendations (Litvan et al., 2012).

### 2.4. Statistical analysis

Statistical analysis was performed using SPSS Statistics 22.0 and Jamovi 2.2.5. Alpha was set at 0.05, but Bonferroni correction was applied for multiple comparisons. For the study of normality, the Shapiro–Wilk test was calculated, and Q-Q plots were examined.

For categorical variables, Pearson’s chi-squared test was calculated for intergroup comparisons. Kruskal-Wallis and *post-hoc* tests were calculated for intergroup comparisons in the case of more than two groups (e.g., AD-MCI, AD-D, and HC). As measure of effect size, eta squared was calculated and regarded as small (*η*^2^ = 0.01), medium (*η*^2^ = 0.06), and large (*η*^2^ = 0.14). Student’s *t*-test or Mann–Whitney *U* test were calculated for two group comparisons (e.g., PD-MCI and HC) and Cohen’s d was reported as effect size, regarded as small (*d* = 0.20), medium (*d* = 0.50), and large (*d* = 0.80). For the study of relationships between quantitative variables, Spearman’s rho correlation was categorized as very low (0–0.29), low (0.30–0.49), moderate (0.50–0.69), high (0.70–0.89) or very high (>0.89). ROC curves were estimated for variables with significant differences between clinical groups and HC and when the area under the curve (AUC) was ≥0.70. Cut-off scores were calculated when AUC ≥0.70 and following Youden’s index (always >0.40). Additionally, sensitivity, specificity, positive predictive value (PPV), negative predictive value (NPV), positive likelihood ratio (LR+), and negative likelihood ratio (LR-) were reported for each cut-off score. To compare the AD-MCI with PD-MCI, ANCOVA models were estimated to control for age, years of education, and sex.

## 3. Results

### 3.1. Alzheimer’s disease: AD-MCI, AD-D, and HC

#### 3.1.1. Intergroup comparisons

We found a group effect in the Objects test, Sun-Moon test Part B, Dots test Part A – time and Dots test Part B with the lowest performance in AD-D. *Post-hoc* analysis revealed differences between AD-MCI and HC in Objects test Part B, Sun-Moon test Part B – errors, and Dots test Part B – errors with large effect sizes. Comparing AD-D and HC, the same differences were found with the addition of Objects test Part A, Sun-Moon test Part B (time and errors), and Dots test Part A – time. By contrast, no group effect was detected for the Sun-Moon test Part A or Dots test Part A – time. Comparisons are shown in Table 2. The main results of all groups are represented in Figure 1. All NN scores are shown in Supplementary material 2 for all clinical groups.

**Table 2:** Intergroup differences on CCD scores for AD and HC groups.

	AD-MCI *N* = 30	AD-D *N* = 30	HC *N* = 30	*H (p)*	η^2^_H_
Objects test					
Part A (/122)	119.7 (2.73)	116.0 (4.94)	120.7 (1.76)	23.801 (<0.001)*^,a, b^	0.25
Part B (/122)	111.3 (7.46)	103.3 (5.98)	119.0 (3.91)	51.56 (<0.001)*^,a, b, c^	0.57
Sun-Moon test					
Part A – time (sec.)	27.23 (8.54)	31.40 (10.70)	24.10 (5.84)	8.73 (0.013)	0.07
Part A – errors (/40)	0.33 (0.71)	0.50 (0.90)	0.03 (0.18)	7.49 (0.024)	0.06
Part B – time (sec.)	39.90 (13.90)	51.72 (23.15)	33.77 (8.75)	14.69 (0.001)*^,b^	0.14
Part B – errors (/40)	2.83 (2.79)	3.21 (3.09)	0.53 (0.94)	20.21 (<0.001)*^,b, c^	0.21
Dots test					
Part A – time (sec.)	34.87 (24.23)	46.66 (25.02)	25.37 (9.67)	14.93 (0.001)*^,b^	0.15
Part A – errors (/9)	0.33 (0.61)	0.38 (0.49)	0.07 (0.25)	7.91 (0.019)	0.07
Part B – time (sec.)	105.87 (60.86)	126.64 (66.12)	70.23 (31.91)	16.84 (<0.001)*^,b, c^	0.17
Part B – errors (/18)	1.27 (1.26)	1.55 (1.44)	0.27 (0.45)	16.14 (<0.001)*^,b, c^	0.16

Objects test Part A/B = true positive + true negative; *significant difference after Bonferroni’s correction; ^a^ *post-hoc* difference between AD-MCI and AD-D; ^b^ *post-hoc* difference between AD-D and HC; ^c^ *post-hoc* difference between AD-MCI and HC.

#### 3.1.2. ROC analysis for group discrimination

In AD-MCI and HC, AUC for Objects test Part B was 0.842 (*p* < 0.001, CI: 0.743–0.941) and cut-off was 120 (sensitivity = 63.33%, specificity = 93.33%, PPV = 90.48%, NPV = 71.79%, LR+ = 18.90, LR− = 0.39, Youden’s index = 0.567). For Sun-Moon test Part B – errors, AUC was 0.759 (*p* = 0.001, CI: 0.635–0.884) and cut-off score was 2 (sensitivity = 60%, specificity = 86.67%, PPV = 81.82%, NPV = 68.42%, LR+ = 9.00 LR− = 0.46, Youden’s index = 0.467). For Dots test Part B – errors AUC was 0.724 (*p* = 0.003, CI: 0.593–0.856) and the cut-off point was 2 (sensitivity = 43.33%, specificity = 100%, PPV = 100%, NPV = 63.83%, LR- = 0.57, Younden’s index = 0.433).

In AD-D and HC, Objects test Part A obtained an AUC of 0.837 (*p* < 0.001, CI: 0.732–0.942) and the optimal cut-off score was 119 (sensitivity = 86.67%, specificity = 70.00%, PPV = 74.29%, NPV = 84%, LR+ = 5.77, LR− = 0.18, Youden’s index = 0.567). AUC for Objects test Part B was 0.983 (*p* < 0.001, CI: 0.957–1.0) and cut-off score was 112 (sensitivity = 96.67%, specificity = 93.33%, PPV = 93.55%, NPV = 96.55%, LR+ = 28.8, LR- = 0.03, Youden’s index = 0.90).

Considering Sun-Moon test Part B, AUCs were 0.731 (*p* = 0.004, CI: 0.595–0.867) and 0.722 (*p* = 0.001, CI: 0.637–0.906) for time and errors, respectively. Cut-off point for time was 38 s (sensitivity = 70%, specificity = 73.33%, PPV = 72.41%, NPV = 70.97%, LR+ = 5.25, LR− = 0.41, Youden’s index = 0.433) and for error was 2 (sensitivity = 63.33%, specificity = 86.67%, PPV = 82.61%, NPV = 70.27%, LR+ = 9.45, LR− = 0.42, Youden’s index = 0.50).

Scores on Dots test Part A – time showed AUC of 0.728 (*p* = 0.005, CI: 0.580–0.875) and its cut-off point was 32 s (sensitivity = 72.41%, specificity = 76.67%, PPV = 75.00%, NPV = 74.19%, LR+ = 6.17, LR− = 0.35, Youden’s index = 0.491). AUC of Dots test Part B – time and errors were 0.819 (*p* < 0.001, CI: 0.705–0.932) and 0.768 (*p* = 0.001, CI: 0.628–0.909) respectively. Cut-off score for time was 85 s (sensitivity = 73.91%, specificity = 76.67% PPV = 70.83%, NPV = 79.31%, LR+ = 6.17, LR− = 0.26, Youden’s index = 0.506) and for errors was 2 (sensitivity = 56.52%, specificity = 100%, PPV = 100%, NPV = 75.00%, LR- = 0.33, Youden’s index = 0.565).

Comparing AD-MCI and AD-D, Objects test Part A showed an AUC of 0.758 (*p* = 0.001, CI: 0.633–0.883) and the cut-off was 119 (sensitivity = 80.00%, specificity = 70.00%, PPV = 72.73%, NPV = 77.78%, LR+ = 5.33, LR− = 0.28, Youden’s index = 0.50). Objects test Part B AUC was 0.801 (*p* < 0.001, CI: 0.686–0.915) and the best cut-off was 108 (sensitivity = 73.33%, specificity = 73.33%, PPV = 73.33%, NPV = 73.33%, LR+ = 5.47, LR− = 0.36, Youden’s index = 0.467).

#### 3.1.3. Convergent validity

Objects test Part A (immediate recognition) showed low correlations with FCSRT scores and low–moderate correlations with ROCF memory tasks. Part B had low–moderate correlations with FCSRT scores and ROCF memory tasks.

Sun-Moon test Part A (naming) showed moderate–high correlations with Stroop – reading and Stroop – naming scores. While the Sun-Moon test Part B was moderately correlated with Stroop–interference score.

**Figure 2:**
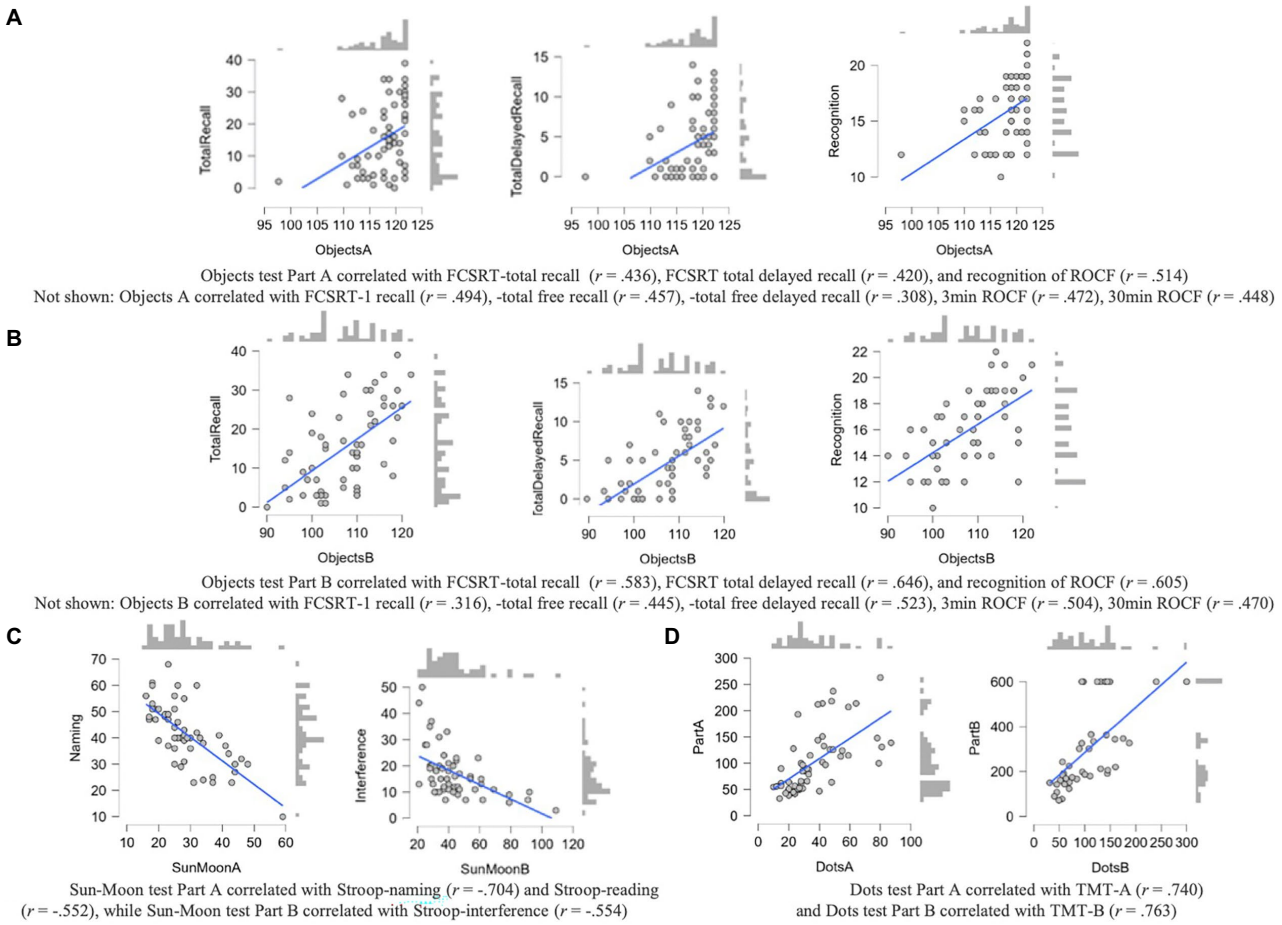
**(A–D)** Correlations between CCD measures and convergent measures of NN in the AD group. All *p* values <0.001. Scores on FCSRT and ROCF were correlated with Objects test Part A scores **(A)** and Objects test Part B scores **(B)**. Scores on Sun-Moon test Part A were correlated with Stroop – word reading scores, while Part B was associated with Stroop – interference **(C)**. Dots test Part A was correlated with TMT-A and Part B with TMT-B **(D)**.

Dots test Part A (one color) showed a high correlation with TMT-A and Part B (two colors) was highly correlated with TMT-B. All correlations are shown in Figures 2A–D.

### 3.2. Parkinson’s disease with mild cognitive impairment and HC

#### 3.2.1. Intergroup comparison

We found a lower performance in the PD-MCI group compared to HC in Objects test Part A and B, Sun-Moon test Part B (time and errors), and Dots test Part B – errors with large effect sizes. Data are shown in Table 3. The main results of all groups are represented in Figure 1.

**Table 3:** Intergroup differences on CCD scores for PD and HC groups.

	PD-MCI *N* = 30	HC *N* = 30	*U* (*p*)	*d*
Objects test				
Part A (/122)	118.4 (3.45)	121.1 (1.61)	221.5 (<0.001)*	1.00
Part B (/122)	110.8 (6.76)	120.0 (3.75)	79.5 (<0.001)*	1.68
Sun-Moon test				
Part A – time (sec.)	30.53 (33.31)	21.27 (6.01)	587.0 (0.042)	0.39
Part A – errors (/40)	0.23 (0.63)	0.03 (0.18)	510.5 (0.085)	0.43
Part B – time (sec.)	51.83 (70.71)	28.63 (8.90)	677.5 (0.001)*	0.46
Part B – errors (/40)	1.73 (2.02)	0.20 (0.48)	687.5 (<0.001)*	1.04
Dots test				
Part A – time (sec.)	32.50 (22.77)	23.07 (11.93)	506.0 (0.180)	0.52
Part A – errors (/9)	0.29 (0.66)	0.07 (0.25)	483.0 (0.101)	0.44
Part B – time (sec.)	82.65 (54.84)	59.47 (52.17)	516.0 (0.038)	0.43
Part B – errors (/18)	0.77 (0.99)	0.10 (0.31)	540.0 (0.002)*	0.91

*Significant difference after Bonferroni’s correction.

#### 3.2.2. ROC analysis for group discrimination

Objects test Part A AUC was 0.751 (*p* = 0.001, CI: 6.22–0.879). Accordingly, cut-off score was 121 (sensitivity = 80.00%, specificity = 70.00%, PPV = 72.73%, NPV = 77.78%, LR+ = 5.33, LR− = 0.28, Youden’s index = 0.50). AUC of Objects test Part B was 0.909 (*p* < 0.001, CI: 0.831–0.986) and its cut-off point was 118 (sensitivity = 83.33%, specificity = 89.66%, PPV = 89.29%, NPV = 83.87%, LR+ = 16.6, LR− = 0.18, Youden’s index = 0.73).

Regarding Sun-Moon test Part B, AUC for time was 0.721 (*p* < 0.005, CI: 0.586–0.855) and for errors was 0.772 (*p* < 0.001, CI: 0.644–0.901) and cut-off points were 37 s (sensitivity = 60%, specificity = 83.33%, PPV = 78.26%, NPV = 67.57%, LR+ = 7.2, LR− = 0.48, Youden’s index = 0.433) and 1 error (sensitivity = 63.33%, specificity = 83.33%, PPV = 79.17%, NPV = 69.44%, LR+ = 7.56, LR− = 0.44, Youden’s index = 0.467).

Dots test Part B – errors showed an AUC value of 0.700 (*p* = 0.014, CI: 0.549–0.835) and its cut-off point was 1 error (sensitivity = 46.15%, specificity = 90%, PPV = 80.00%, NPV = 65.85%, LR+ = 9.2, LR− = 0.42, Youden’s index = 0.362).

#### 3.2.3. Concurrent convergent validity

Objects test Part A showed low–moderate correlations with FCSRT scores and ROCF memory tasks.

Sun-Moon test Part A had moderate–high correlations with Stroop – reading and Stroop – naming. While Sun-Moon test Part B was moderately correlated with Stroop – interference.

**Figure 3:**
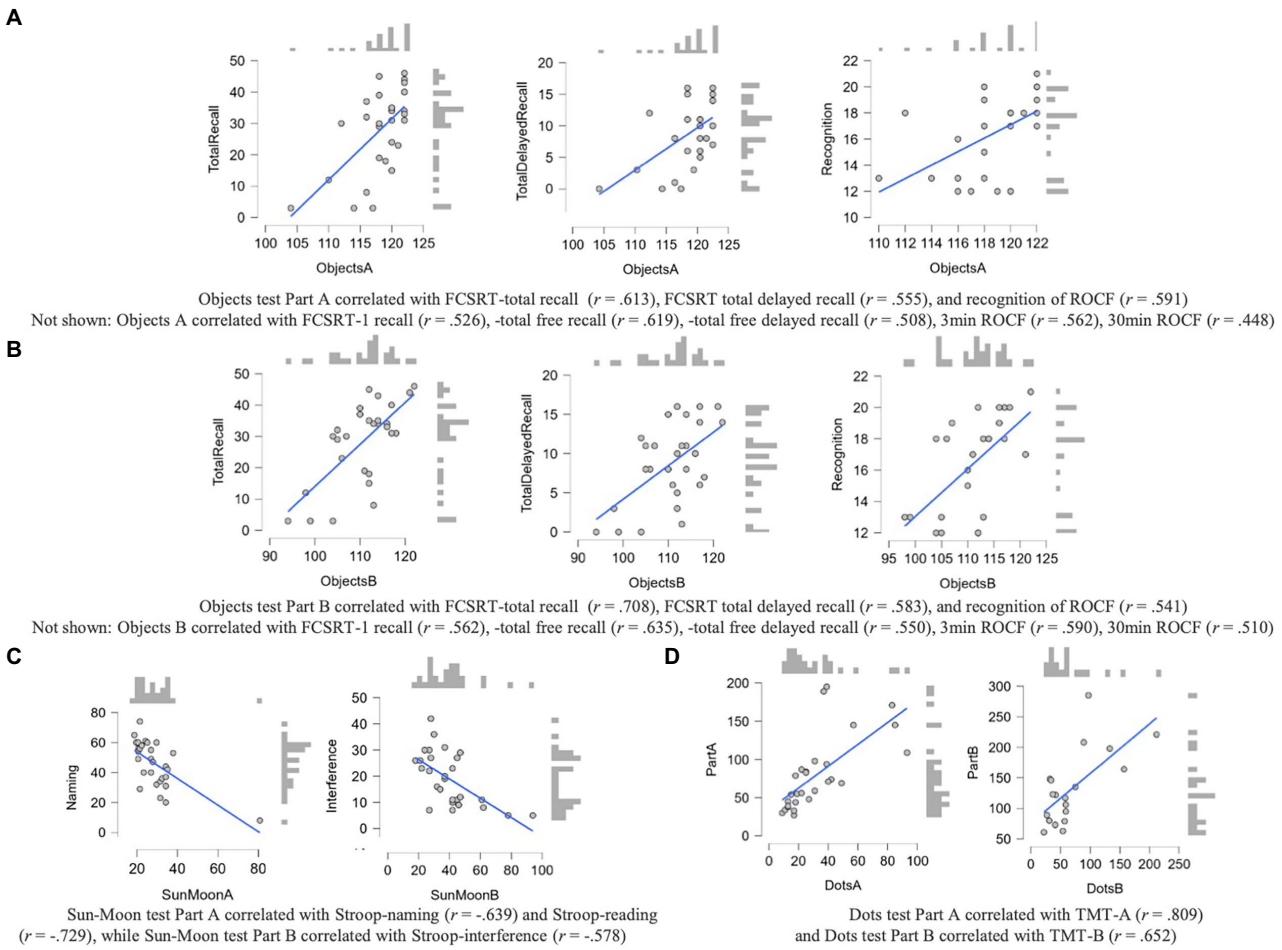
**(A–D)** Correlations between CCD measures and convergent measures of NN in the PD-MCI group. All *p* values <0.001. Scores on FCSRT and ROCF were correlated with Objects test Part A scores **(A)** and Objects test Part B scores **(B)**. Scores on Sun-Moon test Part A were correlated with Stroop – word reading scores, while Part B was associated with Stroop – interference **(C)**. Dots test Part A was correlated with TMT-A and Part B with TMT-B **(D)**.

Dots test Part A correlated highly with TMT-A and Part B moderately with TMT-B. All correlations are shown in Figures 3A–D.

### 3.3. Alzheimer’s disease with MCI and Parkinson’s disease with MCI

Controlling for age, years of education, and sex, ANCOVA showed a worst performance in PD-MCI group compared to AD-MCI group in Sun-Moon test Part A – time (*F* = 6.73, *p* < 0.001, *η*^2^ = 0.337) and Part B – time (*F* = 5.26, *p* = 0.001, *η*^2^ = 0.284) and Dots test Part B – time (*F* = 4.91, *p* = 0.002, *η*^2^ = 0.270) and – errors (*F* = 4.11, *p* = 0.006, *η*^2^ = 0.237).

## 4. Discussion

Cross-cultural neuropsychological instruments are key to avoiding the influence of different confounding variables, such as language, culture, ethnicity, or education level. Although several screening tests have been developed for the assessment of AD and PD, most of them show important limitations for multicultural settings (Jones and Gallo, 2002; Nielsen et al., 2012; Goudsmit et al., 2018). The availability of cross-cultural tests and the specific validation process in the target clinical groups are necessary to improve neuropsychological evaluation.

In this regard, this study aimed to validate the screening tool CCD in AD at different stages of the disease and in PD-MCI. To our knowledge, this is the first validation study of CCD that includes a sample of prodromal stages of AD (supported by biomarkers) and PD with MCI.

In AD, memory measures were especially relevant, showing large effect sizes and high AUCs. Notably, the delayed recognition tasks Objects test Part B showed the largest effect size and the best AUC, even at the early stages of the disease, in accordance with the characteristic cognitive profile of AD with episodic memory deficits (Dubois et al., 2016). We also found differences in the most difficult parts of the Sun-Moon test and Dots test, where executive functions are required, with medium-large effect sizes in AD-D, but also in AD-MCI, supporting the utility of CCD as a screening test at the early stages of the disease, but also as a follow-up measure along the disease.

In our sample of AD participants, the cut-off scores on the Objects test were very similar to the original study of the CCD (Goudsmit et al., 2017), supporting the cross-cultural properties of the test. We found some differences in cut-off of mental speed measures: Sun-Moon test Part B 71 vs. 38 in our sample, Dots test Part A 115 vs. 32 in our study, Part B 216 vs. 85 in our case. However, while the first validation study of CCD included participants with different causes of dementia, we only considered participants with AD, and less impairment was expected compared to other dementias, such as vascular dementia or mixed dementia. Besides, in our group, there were no illiterate participants, differently from the Dutch validation group. Measures of speed are highly correlated with educational level, especially illiteracy (Ardila et al., 2010).

CCD showed correlations with FCSRT, all tasks of ROCF, Stroop, and TMT, supporting the convergent validity of the instrument in AD. Sun-Moon test is based on an interference paradigm similar to the classical Stroop task, which has shown good classification properties between healthy and pathological aging, compared with other executive functions tasks (Guarino et al., 2018). Dots test could be understood as a cross-cultural version of TMT, where differences in colors in part B, instead of number and letter alternation has proven to be useful in non-native English speakers (Kim et al., 2014). The assessment of memory in CCD based on a recognition task of daily life objects without verbal load was remarkable and achieved significant correlations with other episodic memory tasks, which are especially recommended in AD (Gallagher and Koh, 2011).

PD-MCI group showed differences in memory, processing speed, and errors with large effect sizes. While memory deficits were observed on immediate and delayed recognition scores, deficits on errors were related to the most challenging parts of the Sun-Moon and Dots tests, where inhibition is involved. Episodic memory deficits are among the most reported cognitive symptom in PD. In particular, recognition deficits have been described in the literature and associated with a disruption in recollection processes with normal familiarity (Das et al., 2019). In this regard, the recognition tasks of CDD were suitable in the PD-MCI sample and achieved high AUCs, considering that memory was the most impaired domain in our sample. In addition, PD-MCI also shows executive functioning deficits, including inhibition problems (Dirnberger and Jahanshahi, 2013). In CCD, the interference task of the Sun-Moon test and part B of the Dots test showed these deficits by error measures. Only one time measure was statistically significant with the smallest effect size, confirming inhibition problems on executive function scores without a bias of motor limitations.

While the Objects test showed a good balance between sensitivity and specificity values, the time and error cut-off points of the Sun-Moon test and Dots test showed higher values of specificity than sensitivity. In line with AD results, CCD showed good convergent validity properties.

Intriguingly, we found cognitive differences between AD-MCI and PD-MCI, especially in executive functions and speed scores. This suggests the utility of CCD as a screening test in different disorders, not only in AD and related dementias (Delgado-Álvarez et al., 2022). In addition, these results and the high correlations with standardized neuropsychological tests suggest the possibility to explore the usefulness of CCD in the differential diagnosis of different neurodegenerative disorders.

Regarding education, our AD sample showed a low educational level, compared with the Dutch subgroup in the original validation study of CCD: education mean of 5 in native Dutch (range 0 no education – 7 university) vs. 7.10 (AD-MCI) and 7.03 (AD-D) years of education mean in Spaniards (range 0–18) (Goudsmit et al., 2017). The classification properties in our study confirm the utility of CCD in participants with a low level of schooling.

Our study has some limitations. First, we did not include a group of patients with PD cognitively preserved, which would be appropriate to compare with PD-MCI. Second, the level of education in our sample was generally low. Future studies should compare the diagnostic properties between patients with high-and low-education levels. Third, cognitive reserve was not specifically evaluated, although previous studies have reported years of education as a proxy for cognitive reserve (Nicolas et al., 2020; Rosselli et al., 2022).

In conclusion, the CCD showed adequate diagnostic properties for the assessment of patients in early stages of AD and cognitive impairment associated with PD. This confirms the usefulness of CCD as a novel cognitive tool in the assessment of patients with cognitive impairment in different neurological conditions. Furthermore, the similarities in the optimal cut-off scores in the Spanish population in comparison with the previous validation studies support the favorable cross-cultural properties of the test and open the door to conduct collaborative and multicultural studies using the CCD.

## Data availability statement

The raw data supporting the conclusions of this article will be made available by the authors, without undue reservation.

## Ethics statement

The studies involving human participants were reviewed and approved by the Comité de Ética e Investigación Clínica Hospital Clinico San Carlos. The patients/participants provided their written informed consent to participate in this study.

## Author contributions

AD-Á: conceptualization, visualization, data curation, formal analysis, investigation, methodology, writing – original draft, and writing – review and editing. CD-A, MV-S, and MD-C: data curation, investigation, and writing – review and editing. MG, RG-R, MG-M, and MZ-C: investigation and writing – review and editing. JM-G: conceptualization, visualization, funding acquisition, investigation, supervision, and writing – review and editing. JAM-G: conceptualization, visualization, data curation, formal analysis, funding acquisition, investigation, methodology, supervision, writing – original draft, and writing – review and editing. All authors contributed to the article and approved the submitted version.

## Funding

This work was supported by the Instituto de Salud Carlos III (PI19/01260) (co-funded by European Regional Development Fund “A way to make Europe”). JAM-G was supported by the Instituto de Salud Carlos III through the project INT20/00079 (co-funded by European Regional Development Fund “A way to make Europe”). MV-S was supported by the Instituto de Salud Carlos III through a predoctoral contract PFIS (FI20/000145) (co-funded by European Regional Development Fund “A way to make Europe”).

## Conflict of interest

The authors declare that the research was conducted in the absence of any commercial or financial relationships that could be construed as a potential conflict of interest.

## Publisher’s note

All claims expressed in this article are solely those of the authors and do not necessarily represent those of their affiliated organizations, or those of the publisher, the editors and the reviewers. Any product that may be evaluated in this article, or claim that may be made by its manufacturer, is not guaranteed or endorsed by the publisher.
