# Supplementary material for: Validation of the cross-cultural dementia screening test in Alzheimer’s disease and Parkinson’s disease
Source: Front Psychol. 2023 Jan 4;13:1043721. doi: 10.3389/fpsyg.2022.1043721 (PMC9846357; doi:10.3389/fpsyg.2022.1043721)
Supplement: Supplementary file 1 [file Data_Sheet_1.pdf]

Supplementary Material 1. Comparisons between clinical groups and their control groups.

Group	Sex	Age	Education
AD vs. HC	X^2= 1.659, p = .436	F = 0.567, p = .569	H = 1.17, p = .557
PD-MCI vs. HC	X^2 = 3.590, p = .103	t = -1.068, p = .290	U = 568.5, p = .076

Supplementary Material 2. Impaired domain classification based on NN scores.

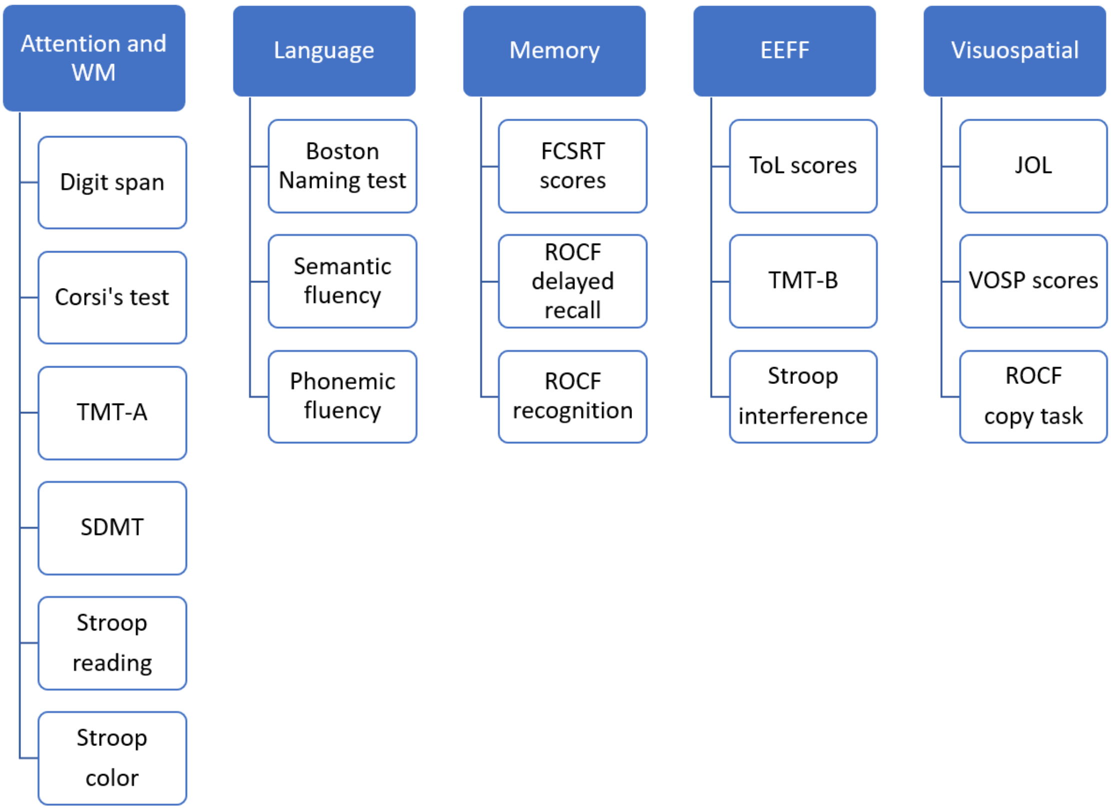

WM = working memory, EEFF = executive functions, SDMT = Symbol Digit Modalities Test, FCSRT = Free and Cued Selective Reminding test, ROCF = Rey-Osterrieth Complex Figure, ToL = Tower of London, TMT = Trail Making Test, JOL = Judgement of Line Orientation, VOSP = Visual Object and Space Perception Battery.

Supplementary Material 3. Performance of patients on tests of the Neuronorma battery.

Test (/score)	AD-MCI		AD-D		PD-MCI	
	Mean	SD	Mean	SD	Mean	SD
Digit span forward (/9)	4.97	0.81	4.73	0.78	5.23	0.90
Digit span backward (/8)	3.27	0.58	3.07	0.74	3.50	0.68
Corsi's test forward (/9)	4.33	0.71	4.17	0.65	4.70	0.84
Corsi's test backward (/9)	3.23	0.73	2.97	0.72	3.70	0.84
TMT – A (seconds)	86.28	48.78	125.00	66.61	79.62	47.48
TMT – B (seconds) [a]	293.25	192.94	335.63	175.30	142.55	74.78
SDMT (/110)	18.62	11.66	9.75	5.81	20.55	11.47
Stroop – word (correct items)	65.86	13.76	58.64	20.53	68.17	24.91
Stroop – color (correct items)	44.10	10.42	38.64	12.70	45.97	15.22
Stroop – interference (correct items)	20.31	11.07	13.44	5.81	18.86	10.21
ToL – total corrects (/10) [b]	2.65	1.90	1.33	1.72	2.63	2.45
ToL – total moves (/200) [b]	44.42	23.27	67.86	30.42	50.28	22.30
ToL – initiation time (seconds) [b]	52.11	20.055	61.71	34.30	58.50	36.36
ToL – execution time (seconds) [b]	430.00	158.14	532.57	159.26	434.61	129.31
ToL – resolution time (seconds) [b]	482.11	169.08	594.29	164.50	461.67	168.79
Boston Naming Test (/60)	42.67	6.51	37.31	5.50	47.72	7.01
Semantic fluency (correct words)	11.67	4.29	9.23	3.57	14.67	5.45
Phonemic fluency (correct words)	8.77	4.36	7.87	3.01	11.23	5.24
FCSRT – 1st recall (/16)	2.80	1.45	1.70	1.51	3.33	1.69
FCSRT – total free recall (/48)	8.67	4.90	4.60	4.32	12.40	6.85
FCSRT – total recall (/48)	20.53	9.43	9.93	9.52	28.33	12.8
FCSRT – delayed free recall (/16)	1.67	2.11	0.30	1.05	3.70	3.45
FCSRT – delayed recall (/16)	6.03	4.12	2.13	3.07	8.87	4.99
ROCF – 3min recall (/36)	17.07	53.18	1.31	2.54	7.34	7.13
ROCF – 30 min recall (/36)	4.07	5.74	0.61	1.33	11.62	24.70
ROCF – recognition task (/24)	17.31	2.79	14.15	2.24	16.52	3.07
Judgment of Line Orientation (/30)	19.25	4.33	15.22	3.06	19.00	5.04
ROCF – copy accuracy (/36)	32.00	7.30	26.96	44.37	27.76	7.94
ROCF – time (seconds)	223.28	89.71	294.54	110.18	222.96	111.25
VOSP – object decision (/20)	16.00	2.01	14.14	2.79	15.30	2.05
VOSP – silhouettes (/20)	12.00	1.41	12.38	1.90	11.23	2.27
VOSP – position discrimination (/20)	19.55	0.68	18.71	1.12	19.20	1.06
VOSP – number localization (/10)	8.48	1.30	7.18	1.36	7.93	1.80

Data are shown as mean and standard deviation.

TMT = Trail Making Test, SDMT = Symbol Digit Modalities Test, ToL= Tower of London, FCSRT = Free and Cued Selective Reminding Test, ROCF = Rey-Osterieth Complex Figure, VOSP = Visual Object and Space Perception battery.

[a] Thirteen participants with AD-D could not finish the task due to cognitive impairment.

[b] Twenty-three participants with AD-D could not finish the task due to cognitive impairment.
